# Replication stress promotes cellular transformation in *Drosophila* epithelium

**DOI:** 10.1038/s41420-025-02383-2

**Published:** 2025-03-12

**Authors:** Maria Molano-Fernández, Ian D. Hickson, Héctor Herranz

**Affiliations:** 1 Department of Cellular and Molecular Medicine, University of Copenhagen, Copenhagen, Denmark; 2 Department of Cellular and Molecular Medicine, Center for Chromosome Stability and Center for Healthy Aging, University of Copenhagen, Copenhagen, Denmark

**Keywords:** Cell division, Cancer models

## Abstract

The accurate control of DNA replication is crucial for the maintenance of genomic stability and cell viability. In this study, we explore the consequences of depleting the replicative DNA Polymerase α (POLA) in the wing disc of *Drosophila melanogaster*. Our findings reveal that reduced POLA activity induces DNA replication stress and activates the replication checkpoint in vivo. Consistent with this, we demonstrate that dATR, a key component in DNA replication checkpoint signaling, is essential for the maintenance of tissue integrity under conditions of compromised POLA activity. We show that cells within the wing disc exhibiting reduced POLA activity arrest in the G2 phase and undergo p53-dependent apoptosis. We also reveal a critical role for DNA Ligase 4 in sustaining cell viability when POLA function is impaired. Most notably, we report the appearance of oncogenic traits in wing disc cells with diminished POLA activity when apoptosis is suppressed. In this context, the overexpression of the oncogene cdc25/string enhances the oncogenic phenotype. These results indicate that a combination of oncogenic activation, replication stress, and suppression of apoptosis is sufficient to promote the emergence of hallmarks of tumorigenesis, highlighting major implications for cancer development in humans.

## Introduction

Genome stability is essential for numerous biological processes [1]. Living organisms are exposed to sources of DNA damage that can cause mutations and genomic instability. In response to DNA damage, cells activate the DNA damage response (DDR). To maintain genome integrity, the DDR coordinates processes such as DNA repair, cell cycle progression, senescence, or apoptosis. Numerous genes associated with the DDR are inactivated in human cancers, underscoring the critical role of the DDR in cancer prevention [2].

During DNA synthesis, the replication machinery encounters obstacles that can slow or stall replication forks and cause DNA damage. This is known as replication stress (RS) and is a hallmark of cancer [3, 4]. The DNA Polymerase α-Primase complex (POLA) initiates DNA synthesis by producing short RNA–DNA primers. On the lagging strand, POLA is periodically required to generate Okazaki fragments [5]. Deficient POLA activity can lead to reduced DNA synthesis and replication fork stalling. Stalled forks can collapse, causing DNA damage and genomic instability [6, 7]. In response to RS, cells activate the replication checkpoint that prevents fork collapse [8–10].

Although analyses in yeast and human cell lines have provided crucial information about the mechanisms and consequences of RS, in vivo analyses in multicellular organisms are still limited. The proliferative epithelium of the larval wing imaginal disc of *Drosophila* is a valuable model to study the cellular responses to RS in vivo. It provides a tractable platform to study cell proliferation, the DDR, and tumor formation in the context of the whole organism [11–14].

Here, we show that depletion of POLA in the wing disc generates RS that is characterized by impaired DNA replication, the accumulation of single-stranded DNA (ssDNA), and checkpoint activation. We demonstrate that cells experiencing RS are eliminated by apoptosis in a p53-dependent manner. We identify dATR and the non-homologous end joining (NHEJ) factor DNA Ligase 4 (Lig4) as key elements maintaining cell viability in cells undergoing RS. We show that cells with RS that are prevented from activating apoptosis accumulate in G2 but progressively exhibit oncogenic traits, including chromosomal instability, loss of epithelial integrity, initiation of tissue invasion, and the expression of malignant markers such as MMP1. Upregulation of the G2/M regulator cdc25/string (stg) circumvents the G2 arrest and enhances the oncogenic phenotype.

## Results

### POLA2 depletion in the wing disc causes replication stress

**Fig. 1:**
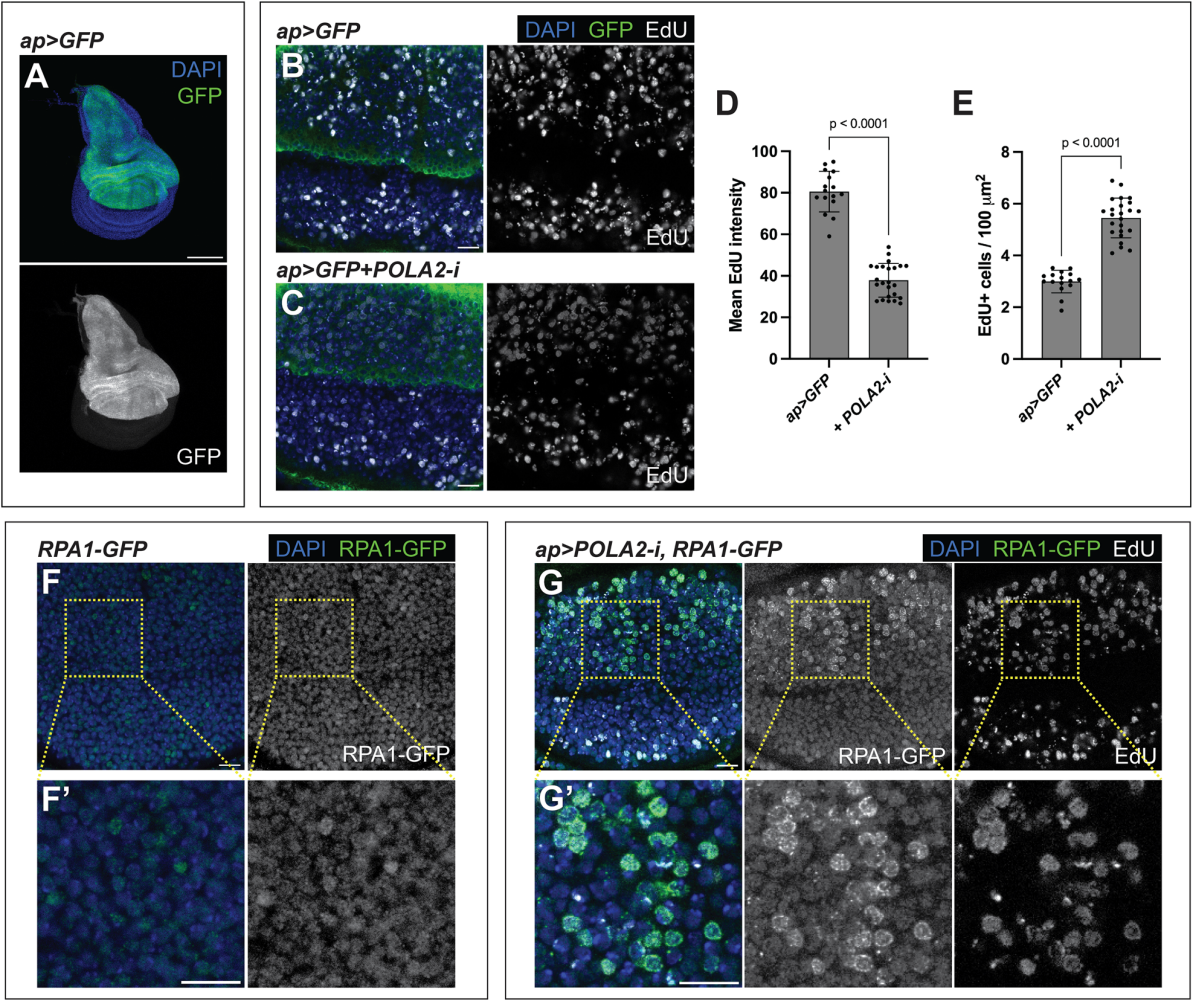
POLA2 downregulation in the wing imaginal disc causes replication stress. **A** Confocal image of a wing imaginal disc expressing GFP under the control of *ap-Gal4*. In the merge, DAPI and GFP are shown in blue and green, respectively. Scale bar, 100 µm. **B, C** Confocal images of the wing pouch of *ap* > *GFP* (**B**) and *ap* > *GFP* + *POLA2-i* (**C**) discs labeled with EdU. In the merge, DAPI, GFP, and EdU are shown in blue, green, and grayscale, respectively. Scale bars, 10 µm. **D, E** Quantification of mean EdU intensity (**D**; *n* = 16, 24) and number of EdU-positive cells per area (**E**; *n* = 16, 24) in the dorsal compartment of the genotypes indicated in **B** and **C**. Data shown are mean ± SD. Statistical analyses were performed using a two-tailed unpaired t-test (**D**) and a two-tailed unpaired t-test with Welch’s correction (**E**). **F** Confocal image of the wing pouch of a wild-type disc carrying an RPA1-GFP sensor. A magnification is shown in **F**'*.* In the merge, DAPI and RPA1-GFP are shown in blue and green, respectively. Scale bars, 10 µm. **G** Confocal image of the wing pouch of an *ap* > *POLA2-i* disc carrying an RPA1-GFP sensor and labeled with EdU. A magnification of the dorsal compartment is shown in **G***'*. In the merge, DAPI, RPA1-GFP, and EdU are shown in blue, green, and grayscale, respectively. Scale bars, 10 µm.

POLA downregulation in human cells slows DNA replication and causes the accumulation of ssDNA, which are hallmarks of RS [4, 6, 9, 15]. The *Drosophila* POLA complex is composed of POLA1, POLA2, Prim1, and Prim2. To compromise the function of the POLA complex, we employed POLA2-RNAi (POLA2-i) transgenes (Fig S1). We employed the Gal4/UAS/Gal80ts system to deplete POLA2 in a spatially and temporally controlled manner [16]. We utilized apterous-Gal4 (ap-Gal4), which is active in the dorsal compartment of the wing disc [17], allowing the use of the ventral compartment as an internal control (Fig. 1A).

Reduced DNA replication fork speed is characteristic of RS [4]. EdU can be used to label cells synthesizing DNA during S-phase. Cells expressing POLA2-i (GFP-positive) showed lower levels of EdU as compared to cells in the otherwise normal ventral compartment (GFP-negative) (Fig. 1B–D). Although the EdU intensity was reduced, the number of EdU-positive cells was higher in discs expressing POLA2-i than in control tissues (Fig. 1B, C, E), suggesting that cells downregulating POLA2 spend more time in S-phase than controls. These results confirm that POLA2 depletion in the wing disc slows DNA replication.

POLA depletion can uncouple leading and lagging strand synthesis, causing an accumulation of ssDNA, which is another hallmark of RS [4, 6]. Compared to double-stranded DNA (dsDNA), ssDNA is relatively unstable, rendering it susceptible to attack by nucleases. To counteract this, cells rely on Replication Protein A (RPA), which coats and protects ssDNA [18]. RPA is commonly used as a marker of ssDNA. Basal levels of an RPA1-GFP transgene [19] were present in normal wing imaginal disc cells (Fig. 1F, F’). Depletion of POLA2 resulted in the formation of RPA1-GFP foci, which were absent in the otherwise normal ventral compartment. The cells with RPA1-GFP foci were positive for EdU, indicating that POLA2-depleted cells accumulate ssDNA in S-phase (Fig. 1G, G’). These observations demonstrate that POLA2 downregulation slows DNA replication and causes an increase in ssDNA, which are central hallmarks of RS [4].

### POLA2 downregulation causes replication checkpoint activation

Defects in DNA are sensed by the DDR. The conserved Ataxia-Telangiectasia Mutated (ATM) and the Ataxia Telangiectasia and Rad3-related (ATR) kinases are the primary sensors of DNA damage. ATM and ATR largely respond to different DNA lesions. While ATM responds primarily to double-strand breaks (DSBs), ATR mediates the replication checkpoint and is activated by stalled forks or increased ssDNA [20, 21]. We will refer to the *Drosophila* ATM and ATR orthologues as dATM and dATR, respectively [22].

**Fig. 2:**
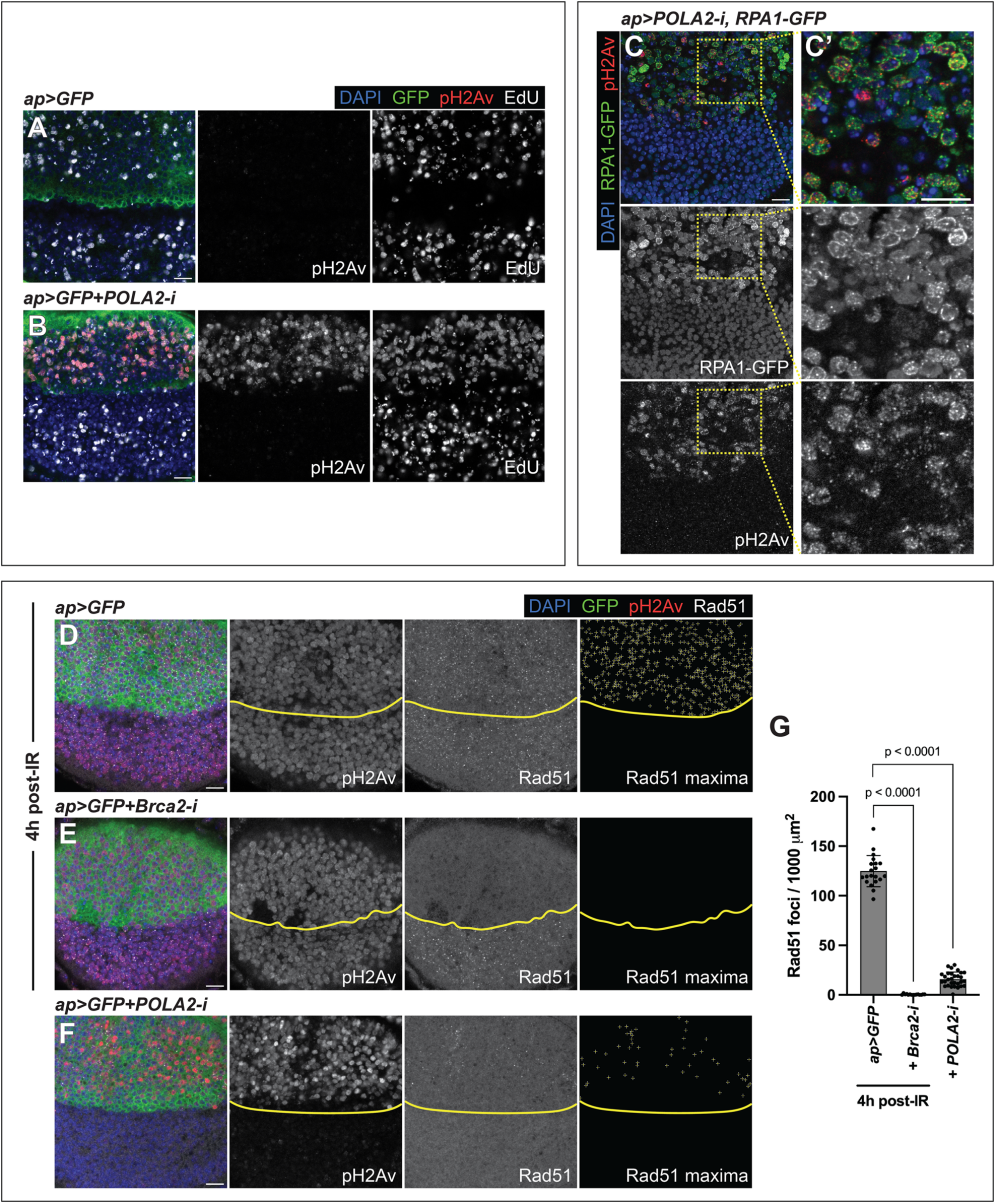
Activation of the replication checkpoint upon POLA2 depletion. **A, B** Confocal images of the wing pouch of *ap* > *GFP* (**A**) and *ap* > *GFP* + *POLA2-i* (**B**) discs labeled with anti-pH2Av and EdU. In the merge, DAPI, GFP, pH2Av, and EdU are shown in blue, green, red, and grayscale, respectively. Scale bars, 10 µm. **C** Confocal image of the wing pouch of an *ap* > *POLA2-i* disc carrying an RPA1-GFP sensor and labeled with anti-pH2Av. A magnification of the dorsal compartment is shown in **C***'*. In the merge, DAPI, RPA1-GFP, and pH2Av are shown in blue, green, and red, respectively. Scale bars, 10 µm. **D–F** Confocal images of the wing pouch of *ap* > *GFP* (**D**) and *ap* > *GFP+Brca2-i* (**E**) discs 4 h after irradiation and of a non-irradiated *ap* > *GFP* + *POLA2-i* (**F**) disc stained with anti-pH2Av and anti-Rad51. The DV boundary is outlined in yellow. The Rad51 maxima panel exhibits the selection of Rad51 foci in the dorsal compartment. In the merge, DAPI, GFP, pH2Av, and Rad51 are shown in blue, green, red, and grayscale, respectively. Scale bars, 10 µm. **G** Quantification of the Rad51 foci per area in the dorsal compartment of the genotypes indicated in *D-F* (*n* = 20, 15, 30). Data shown are mean ± SD. Statistical analyses were performed using a two-tailed Mann–Whitney test (for the *ap* > *GFP vs ap* > *GFP+Brca2-i* comparison) and a two-tailed unpaired t-test with Welch’s correction (for the *ap* > *GFP vs ap* > *GFP* + *POLA2-i* comparison).

Active ATM and ATR phosphorylate numerous substrates to coordinate DNA repair with cell cycle progression. One of these substrates is histone H2AX (H2Av in *Drosophila* [23]). Antibodies against phosphorylated H2Av (pH2Av) are used as markers of checkpoint activation in *Drosophila*. POLA2-depleted discs exhibited pH2Av signal specifically in cells with RS, as revealed by reduced EdU and increased RPA1-GFP (Fig. 2A–C’; Fig S2). This indicates checkpoint activation. Depletion of Prim2, another POLA complex member, led to a similar outcome (Fig S3). Checkpoint activation in S-phase suggests that the dATR-dependent replication checkpoint was activated. However, H2AX can also be phosphorylated by ATM in response to DSBs [24–26]. To distinguish between both possibilities, we analyzed the formation of Rad51 foci. Rad51 mediates homologous recombination (HR), a critical pathway for repairing DSBs in the S- and G2-phases [27]. Rad51 accumulates in DNA repair foci after genotoxic stress, such as ionizing radiation (IR) [28]. Wing discs exposed to IR exhibited Rad51 foci and pH2Av signal (Fig. 2D, G; Fig S4). Brca2 is involved in HR and is required for the loading of Rad51 onto DSBs [29, 30]. To confirm that the Rad51 foci corresponded to centers of HR, we depleted Brca2, exposed those discs to IR, and labeled them with anti-Rad51. Rad51 foci were absent in Brca2-depleted tissues exposed to IR (Fig. 2E, G; Fig S4), confirming that the Rad51 foci correspond to HR centers and mark DSBs. We quantified Rad51-positive foci in discs exposed to IR and POLA2-depleted discs. Although the pH2Av levels were comparable in both contexts, discs exposed to IR presented more Rad51 foci than discs downregulating POLA2 (Fig. 2D, F, G; Fig S4). These results indicate that, although POLA2-depleted cells are positive for pH2Av, only a small proportion of those cells show signs of DSBs (Rad51-positive). These data suggest that downregulation of POLA2 predominantly triggers the activation of the replication checkpoint in vivo. Normal wing discs did not exhibit pH2Av signal or Rad51 foci (Fig. S4).

### POLA2 downregulation causes p53-dependent apoptosis

**Fig. 3:**
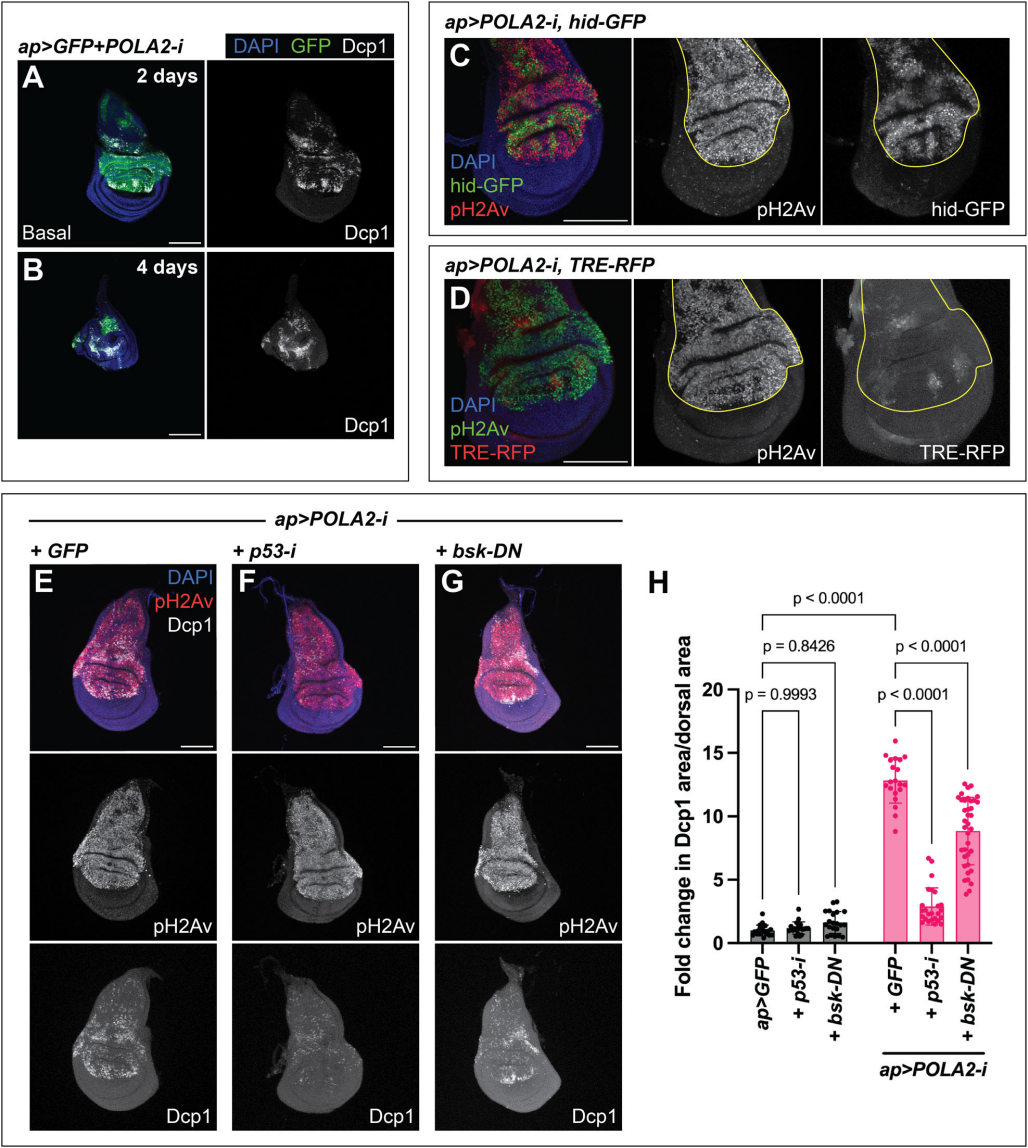
Regulation of apoptosis in imaginal discs with POLA2 knockdown. **A**, **B** Confocal images of *ap* > *GFP* + *POLA2-i* wing imaginal discs stained with anti-Dcp1. Transgene expression was induced for either 2 days (**A**) or 4 days (**B**). The basal side of the disc is displayed in **A**. In the merge, DAPI, GFP, and Dcp1 are shown in blue, green, and grayscale, respectively. Scale bars, 100 µm. **C** Confocal image of an *ap* > *POLA2-i* disc expressing hid-GFP and stained with anti-pH2Av. The dorsal compartment is outlined in yellow. In the merge, DAPI, hid-GFP, and pH2Av are shown in blue, green, and red, respectively. Scale bar, 100 µm. **D** Confocal image of an *ap* > *POLA2-i* disc expressing TRE-RFP and stained with anti-pH2Av. The dorsal compartment is outlined in yellow. In the merge, DAPI, pH2Av, and TRE-RFP are shown in blue, green, and red, respectively. Scale bar, 100 µm. **E-G** Confocal images of *ap* > *POLA2-i* + *GFP* (GFP not shown) (**E**), *ap* > *POLA2-i* + *p53-i* (**F**), and *ap* > *POLA2-i+bsk-DN* (**G**) discs stained with anti-pH2Av and anti-Dcp1. In the merge, DAPI, pH2Av, and Dcp1 are shown in blue, red, and grayscale, respectively. Scale bars, 100 µm. **H** Quantification of apoptotic area per dorsal area represented as a fold change normalized to control discs (*ap* > *GFP*). The analyzed genotypes were *ap* > *GFP* (*n* = 20), *ap* > *GFP* + *p53-i* (*n* = 20), *ap* > *GFP+bsk-DN* (*n* = 21), and the three genotypes indicated in **E**–**G** (*n* = 20, 25, 37). Note that the data points for *ap* > *GFP* and *ap* > *POLA2-i* + *GFP* are the same as in Fig. 5G and Fig. 6G because these experiments were run in parallel. Data shown are mean ± SD. Statistical analysis was performed using a two-way ANOVA followed by a Tukey’s HSD test. Only the relevant one-to-one comparisons are shown.

Apoptosis is activated in response to different forms of stress to eliminate harmful cells and maintain tissue integrity [31]. We observed that discs expressing POLA2-i were labeled by the apoptotic marker Dcp1 [32] (Fig. 3A, B). These discs showed a gradual loss of viability, and depletion of POLA2 for 4 days led to a near elimination of the tissue expressing POLA2-i (GFP-positive) (Fig. 3B).

p53 and the c-Jun N-terminal protein kinase (JNK) are involved in the induction of apoptosis in response to DNA damage [33–38]. We analyzed whether those pathways were active and contributed to the elimination of cells with RS induced by POLA2-i. POLA2 downregulation caused a robust induction of the reporter hid-GFP (Fig. 3C), which includes a p53 response element and serves as a surrogate of p53 transcriptional activity [39, 40]. Importantly, expression of p53-i in discs with reduced POLA2 led to a dramatic decrease in the apoptotic levels (Fig. 3E, F, H). Discs depleted of POLA2 showed a mild increase in the expression of TRE-RFP, a transgenic reporter of JNK activity that contains binding sites for the JNK transcription factor AP1 [41] (Fig. 3D). JNK downregulation by expression a dominant negative version of the *Drosophila* JNK orthologue basket (bsk-DN) in discs expressing POLA2-i (POLA2-i+bsk-DN) led to a mild reduction in the apoptotic signal as compared with POLA2-i+GFP discs (Fig. 3E, G, H). Expression of p53-i or bsk-DN on their own did not have any major effect on tissue growth or apoptosis (Fig. 3H; Fig. S5). Together, these observations imply that p53 plays a central function in the response to RS, and that JNK might play a subsidiary role in the elimination of those cells by apoptosis.

### POLA2 knockdown results in G2 arrest

**Fig. 4:**
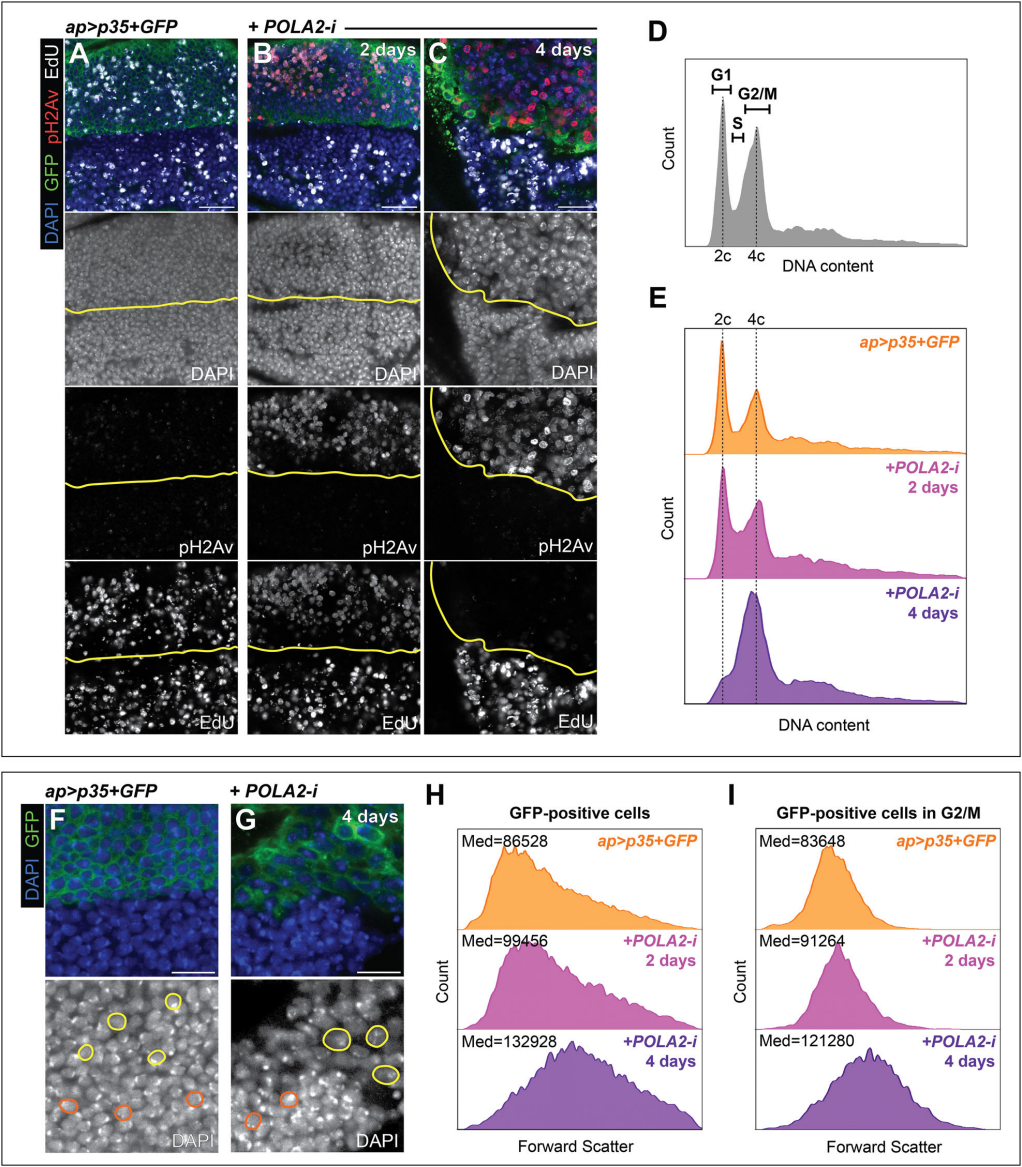
Cell cycle analysis of imaginal discs downregulating POLA2. **A-C** Confocal images of the wing pouch of *ap* > *p35* + *GFP* discs (**A**) and *ap* > *p35* + *GFP* + *POLA2-i* discs induced for either 2 days (**B**) or 4 days (**C**) stained with anti-pH2Av and EdU. The DV boundary is outlined in yellow. In the merge, DAPI, GFP, pH2Av, and EdU are shown in blue, green, red, and grayscale, respectively. Scale bars, 10 µm. **D** DNA content profile obtained with flow cytometry of cells from wild-type wing imaginal discs (*n* = 37164). **E** DNA content profiles obtained with flow cytometry of dorsal (GFP-positive) cells from *ap* > *p35* + *GFP* discs (*n* = 19935) and *ap* > *p35* + *GFP* + *POLA2-i* discs induced for either 2 days (*n* = 16717) or 4 days (*n* = 14482). **F, G** Confocal images of the wing pouch of an *ap* > *p35* + *GFP* disc (**F**) and an *ap* > *p35* + *GFP* + *POLA2-i* disc induced for 4 days (**G**). Examples of cells from the dorsal (GFP-positive) and ventral (GFP-negative) compartments are outlined in yellow and orange, respectively. In the merge, DAPI and GFP are shown in blue and green, respectively. Scale bars, 10 µm. **H, I** Forward scatter profiles obtained with flow cytometry of cells from the dorsal compartment (GFP-positive) (**H**) and cells from the dorsal compartment that are specifically in G2/M (**I**). The genotypes analyzed were *ap* > *p35* + *GFP* (*n* = 19935 in **H**, 5042 in **I**) and *ap* > *p35* + *GFP* + *POLA2-i* induced for either 2 days (*n* = 16717 in **H**, 4124 in **I**) or 4 days (*n* = 14482 in **H**, 5374 in **I**). The median (Med) forward scatter value for each specific genotype is indicated at the top left of each panel. In this figure, *ap* > *p35* + *GFP* + *POLA2-i* discs with induction lengths of 4 days spent 7 days at 18 °C prior to induction.

We investigated whether RS affected cell cycle progression. Given that cell viability is compromised after POLA2 depletion, we suppressed apoptosis by expressing the baculovirus caspase inhibitor p35 [42]. We performed EdU labeling in POLA2-i + p35 discs dissected 2 and 4 days after transgene expression. Control discs showed a similar EdU signal in dorsal and ventral cells (Fig. 4A). Discs expressing POLA2-i and p35 for 2 days exhibited pH2Av signal and a reduction in EdU (Fig. 4B). Discs analyzed 4 days after the expression of POLA2-i and p35 ceased to incorporate EdU, indicating cell cycle arrest (Fig. 4C). To determine the cell cycle phase at which cells were arrested, we employed DNA content analysis by flow cytometry in POLA2-i + p35 discs. Discs dissected 2 days after transgene expression revealed an increase in the proportion of cells in S-phase. Discs dissected 4 days after transgene expression showed an accumulation of cells with a 4c content, indicative of G2 arrest (compare Fig. 4E, with control discs showed in Fig. 4D).

Visual inspection of cells co-expressing POLA2-i and p35 suggested that these cells were larger than control cells (Fig. 4F, G). We quantified cell size by analysis of the forward scatter value obtained with flow cytometry and observed that cells co-expressing POLA2-i and p35 analyzed 2 days after transgene induction were, on average, bigger than control cells. Cells analyzed 4 days after transgene induction were even larger (Fig. 4H). Cells in G2 are typically larger than cells in G1. Therefore, the accumulation in G2 in POLA2-i + p35 discs could account for the increase in observed cell size. To test this, we obtained forward scatter values from cells in G2 only (4c content) and found that those cells increased gradually in size after POLA2 depletion as compared to control cells in G2 (Fig. 4I). This observation suggests a global increase in cell size independent of the phase of the cell cycle in which these cells reside.

### dATR maintains tissue integrity in discs with reduced POLA2

**Fig. 5:**
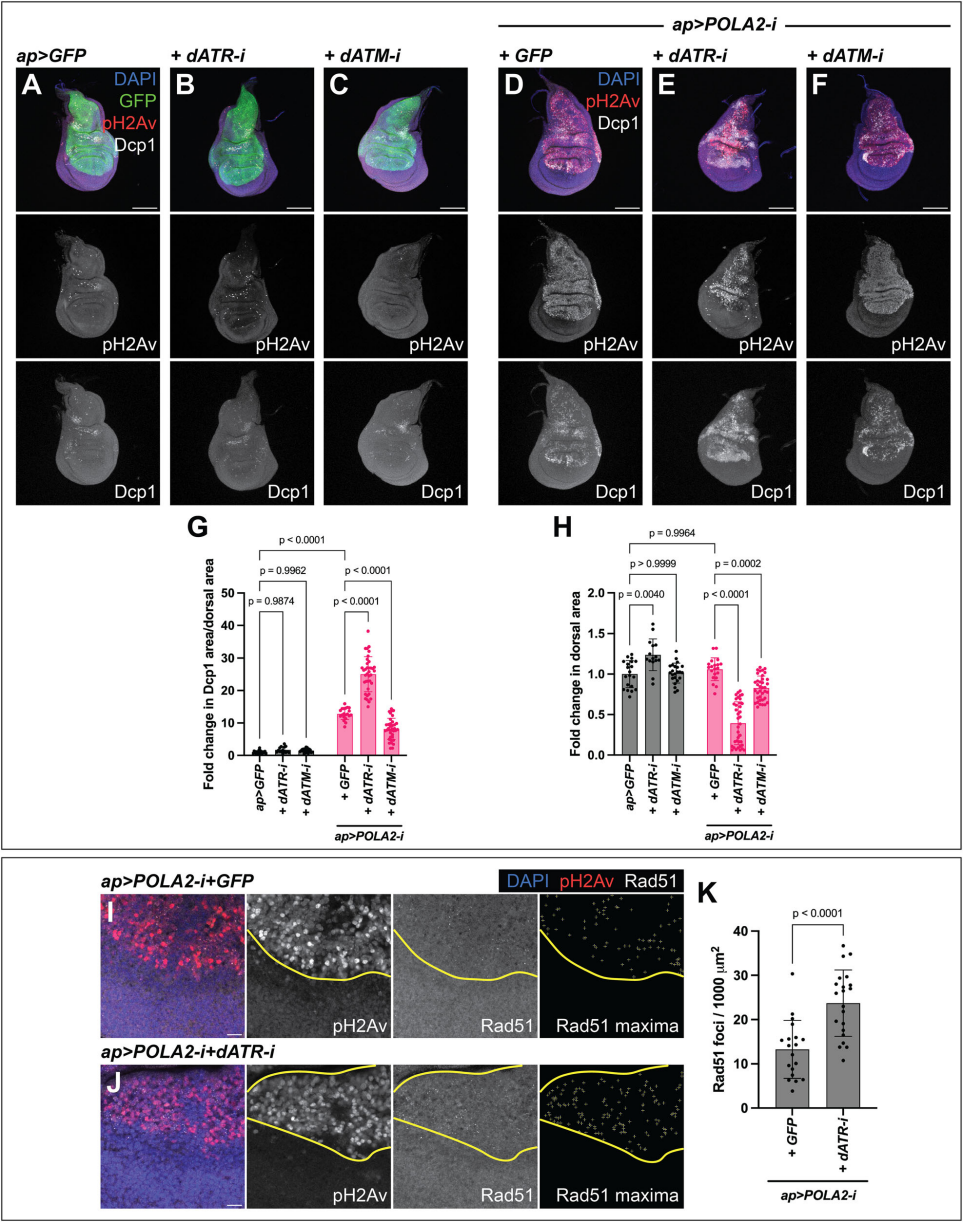
dATR sustains tissue integrity in imaginal discs with reduced POLA2. **A-C** Confocal images of *ap* > *GFP* (**A**), *ap* > *GFP* + *dATR-i* (**B**), and *ap* > *GFP* + *dATM-i* (**C**) discs stained with anti-pH2Av and anti-Dcp1. In the merge, DAPI, GFP, pH2Av, and Dcp1 are shown in blue, green, red, and grayscale, respectively. Scale bars, 100 µm. **D-F** Confocal images of *ap* > *POLA2-i* + *GFP* (GFP not shown) (**D**), *ap* > *POLA2-i* + *dATR-i* (**E**), and *ap* > *POLA2-i* + *dATM-i* (**F**) discs stained with anti-pH2Av and anti-Dcp1. In the merge, DAPI, pH2Av, and Dcp1 are shown in blue, red, and grayscale, respectively. Scale bars, 100 µm. **G, H** Quantification of apoptotic area per dorsal area (**G**) and of dorsal area (**H**) of the genotypes indicated in **A**–**F** (*n* = 20, 15, 23, 20, 37, 37) represented as a fold change normalized to control discs (*ap* > *GFP*). Note that the data points in **G** for *ap* > *GFP* and *ap* > *POLA2-i* + *GFP* are the same as in Fig. 3H and Fig. 6G, and the data points in **H** are the same as in Fig. 6H because these experiments were run in parallel. Data shown are mean ± SD. Statistical analysis was performed using a two-way ANOVA followed by a Tukey’s HSD test. Only the relevant one-to-one comparisons are shown. **I, J** Confocal images of the wing pouch of *ap* > *POLA2-i* + *GFP* (GFP not shown) (**I**) and *ap* > *POLA2-i* + *dATR-i* (**J**) discs labeled with anti-pH2Av and anti-Rad51. The DV boundary and outer border of the wing pouch are outlined in yellow. The Rad51 maxima panel exhibits the selection of Rad51 foci in the dorsal compartment. In the merge, DAPI, pH2Av, and Rad51 are shown in blue, red, and grayscale, respectively. Scale bars, 10 µm. **K** Quantification of the Rad51 foci per area in the dorsal compartment of the genotypes indicated in **I** and **J** (*n* = 19, 21). Note that the data points for *ap* > *POLA2-i* + *GFP* are the same as in Fig. 6K because these experiments were run in parallel. Data shown are mean ± SD. Statistical analysis was performed using a two-tailed unpaired t-test.

dATM and dATR play major roles controlling tissue integrity in cells with IR-induced DNA damage [14, 22, 43, 44]. However, their roles in *Drosophila* tissues experiencing RS have not been defined in detail. We measured levels of apoptosis and tissue size as indicators of tissue integrity. We did not observe an increase in the levels of apoptosis or defects in tissue size in discs expressing either dATM-i or dATR-i on their own (Fig. 5A–C, G, H). Notably, POLA2-i+dATR-i discs exhibited a robust increase in the levels of apoptosis and a concomitant reduction in tissue size, as compared to POLA2-i+GFP discs (Fig. 5D, E, G, H). In contrast, POLA2-i+dATM-i discs did not show an increase in the levels of apoptosis and only resulted in a mild reduction in tissue size (Fig. 5D, F, G, H). Collectively, these observations indicate that dATR sustains cell viability and tissue development in wing imaginal discs with RS. In contrast, dATM appears to play a rather minor function in this context. Our results are consistent with previous observations obtained in *Drosophila* showing that dATR mutants cause an increase in apoptosis in POLA1 heterozygous discs [45].

ATR prevents replication fork collapse and consequent DNA damage in human cell lines with RS [6, 8, 46]. Given that DNA damage in the wing disc induces apoptosis [34, 36–38, 47], we reasoned that dATR might sustain cell viability by preventing the accumulation of DNA damage in discs with RS. We analyzed if POLA2-i+dATR-i discs showed increased DNA damage as compared to POLA2-i+GFP discs. We observed a significant increase in the number of Rad51 foci in POLA2-i+dATR-i discs as compared to POLA2-i+GFP discs (Fig. 5I–K; Fig. S6), indicating that dATR limits the accumulation of DNA damage in discs with RS.

### Lig4 sustains cell viability in tissues with reduced POLA2

DNA damage in cells experiencing RS correlates with reduced cell viability (Fig. 5). This implies that proteins involved in DNA repair might limit the accumulation of DNA damage and thus sustain cell survival. We studied the involvement of the error-free HR and the error-prone NHEJ pathways in this process [48].

**Fig. 6:**
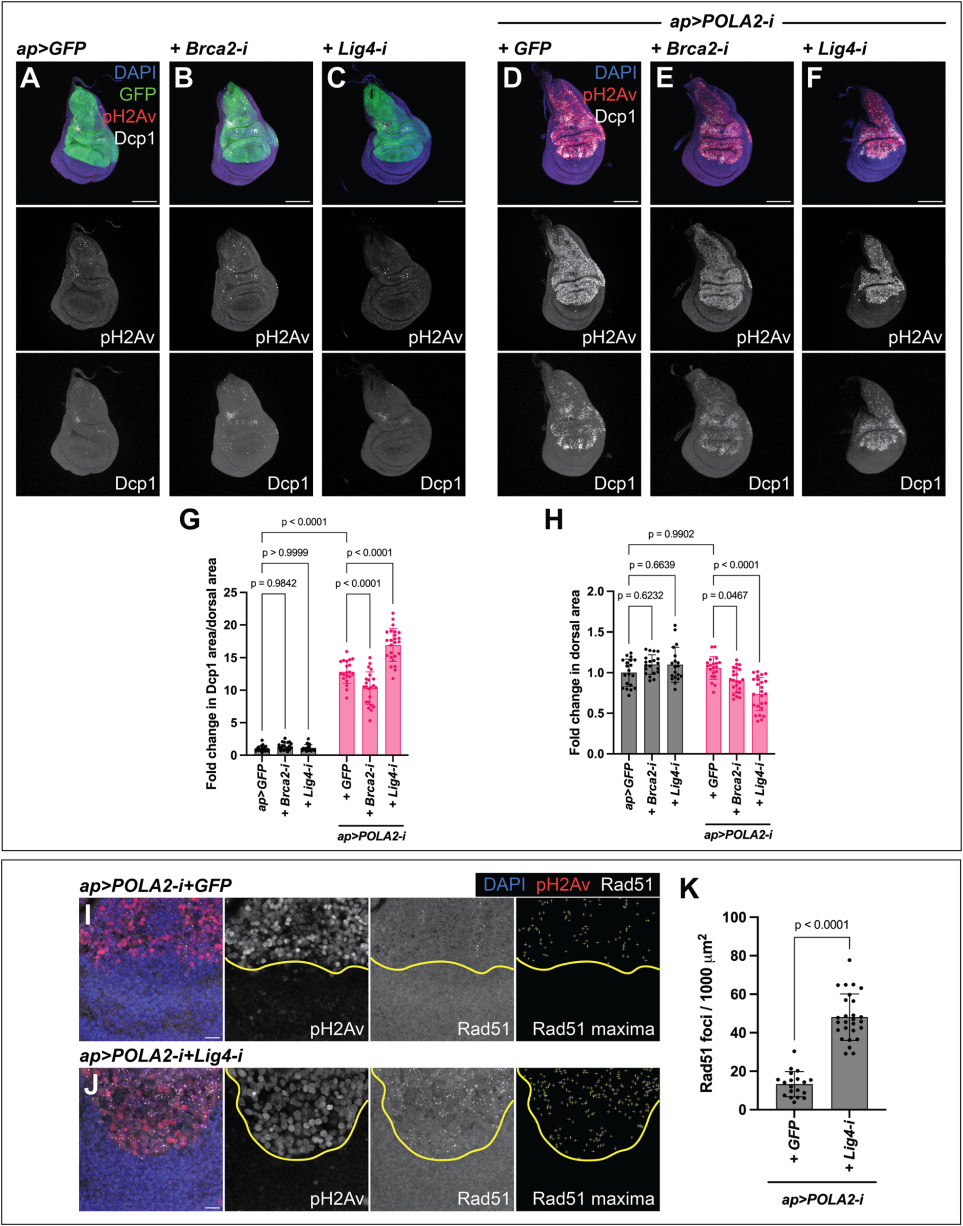
Lig4 supports tissue viability in imaginal discs with reduced POLA2. **A-C** Confocal images of *ap* > *GFP* (**A**), *ap* > *GFP+Brca2-i* (**B**), and *ap* > *GFP+Lig4-i* (**C**) discs stained with anti-pH2Av and anti-Dcp1. In the merge, DAPI, GFP, pH2Av, and Dcp1 are shown in blue, green, red, and grayscale, respectively. Scale bars, 100 µm. **D-F** Confocal images of *ap* > *POLA2-i* + *GFP* (GFP not shown) (**D**), *ap* > *POLA2-i+Brca2-i* (**E**), and *ap* > *POLA2-i+Lig4-i* (**F**) discs stained with anti-pH2Av and anti-Dcp1. In the merge, DAPI, pH2Av, and Dcp1 are shown in blue, red, and grayscale, respectively. Scale bars, 100 µm. **G, H** Quantification of apoptotic area per dorsal area (**G**) and of dorsal area (**H**) of the genotypes indicated in **A**–**F** (*n* = 20, 20, 19, 20, 23, 25) represented as a fold change normalized to control discs (*ap* > *GFP*). Note that the data points in *G* for *ap* > *GFP* and *ap* > *POLA2-i* + *GFP* are the same as in Fig. 3H and Fig. 5G, and the data points in **H** are the same as in Fig. 5H because these experiments were run in parallel. Data shown are mean ± SD. Statistical analysis was performed using a two-way ANOVA followed by a Tukey’s HSD test. Only the relevant one-to-one comparisons are shown. **I, J** Confocal images of the wing pouch of *ap* > *POLA2-i* + *GFP* (GFP not shown) (**I**) and *ap* > *POLA2-i+Lig4-i* (**J**) discs labeled with anti-pH2Av and anti-Rad51. The DV boundary is outlined in yellow. The Rad51 maxima panel exhibits the selection of Rad51 foci in the dorsal compartment. In the merge, DAPI, pH2Av, and Rad51 are shown in blue, red, and grayscale, respectively. Scale bars, 10 µm. **K** Quantification of the Rad51 foci per area in the dorsal compartment of the genotypes indicated in **I** and **J** (*n* = 19, 27). Note that the data points for *ap* > *POLA2-i* + *GFP* are the same as in Fig. 5K because these experiments were run in parallel. Data shown are mean ± SD. Statistical analysis was performed using a two-tailed unpaired t-test with Welch’s correction.

We depleted Brca2 to inhibit HR. Expression of Brca2-i alone did not result in the induction of apoptosis, nor did it lead to an alteration in disc size (Fig. 6A, B, G, H). POLA2-i+Brca2-i discs showed a mild reduction in tissue size compared with POLA2-i+GFP discs. Surprisingly, the levels of apoptosis were also mildly reduced (Fig. 6D, E, G, H). Our results indicate that Brca2 does not play a major role in sustaining cell viability and tissue integrity in cells with reduced POLA2 activity.

To interrogate the role of NHEJ, we downregulated Lig4, a crucial element mediating the final ligation step in NHEJ [49]. Lig4 downregulation did not have a major impact in wing imaginal discs (Fig. 6A, C, G, H). However, POLA2-i+Lig4-i discs showed a robust increase in the levels of apoptosis accompanied by a reduction in tissue size as compared to POLA2-i+GFP discs (Fig. 6D, F, G, H). These data show that, although downregulation of Lig4 did not have an overt impact on imaginal discs, it led to a decay in tissue viability when combined with POLA2 downregulation.

Lig4 is involved in repairing DSBs [48]. We reasoned that Lig4 could act in cells with RS to limit the accumulation of unrepaired DNA damage. Consistently, we observed a robust increase in the number of Rad51 foci in POLA2-i+Lig4-i discs, as compared with POLA2-i+GFP discs (Fig. 6I–K; Fig. S7). These results reveal a central role of Lig4 in sustaining genomic stability and cell viability in discs with RS in vivo.

### POLA2 prevents tumorigenesis

**Fig. 7:**
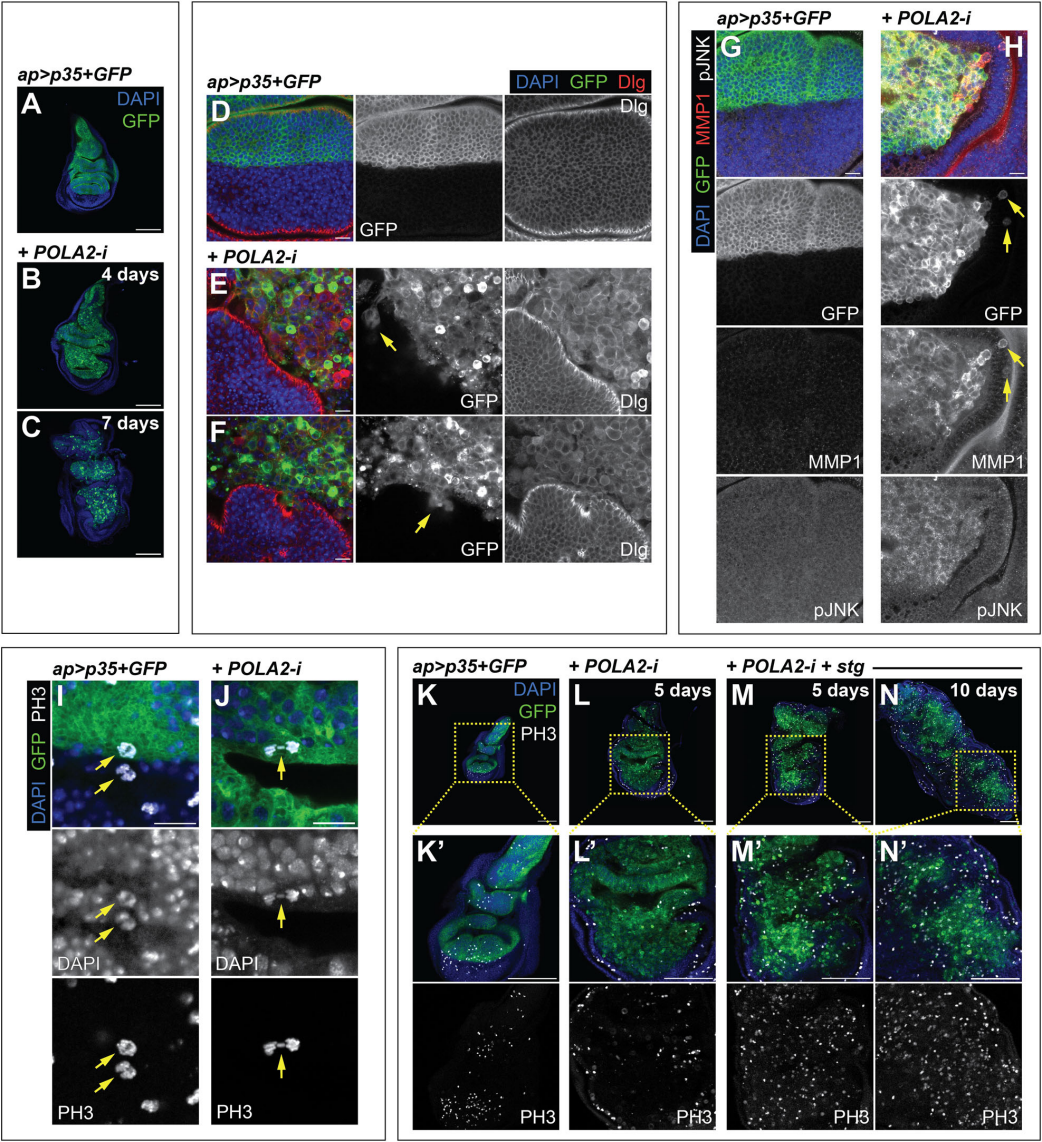
Oncogenic traits in tissues with POLA2 downregulation and apoptosis suppression. **A-C** Confocal images of *ap* > *p35* + *GFP* discs (**A**) and *ap* > *p35* + *GFP* + *POLA2-i* discs induced for either 4 days (**B**) or 7 days (**C**). DAPI and GFP are shown in blue and green, respectively. Scale bars, 100 µm. **D-F** Confocal images of *ap* > *p35* + *GFP* discs (**D**) and *ap* > *p35* + *GFP* + *POLA2-i* discs induced for 7 days (**E**, **F**) stained with anti-Dlg. Yellow arrows indicate clusters of GFP-positive cells that appear to detach from the main GFP-positive tissue. In the merge, DAPI, GFP, and Dlg are shown in blue, green, and red, respectively. Scale bars, 10 µm. **G, H** Confocal images of the wing pouch of *ap* > *p35* + *GFP* (**G**) and *ap* > *p35* + *GFP* + *POLA2-i* (**H**) discs induced for 4 days and stained with anti-MMP1 and anti-pJNK. Yellow arrows indicate dorsal GFP-positive cells that are located in the ventral compartment. In the merge, DAPI, GFP, MMP1, and pJNK are shown in blue, green, red, and grayscale, respectively. Scale bars, 10 µm. **I, J** Confocal images of mitotic cells (yellow arrows) stained with anti-PH3 in *ap* > *p35* + *GFP* discs (**I**) and *ap* > *p35* + *GFP* + *POLA2-i* discs induced for 5 days (**J**). In the merge, DAPI, GFP, and PH3 and shown in blue, green, and grayscale, respectively. Scale bars, 10 µm. **K-N** Confocal images of *ap* > *p35* + *GFP* discs (**K**), *ap* > *p35* + *GFP* + *POLA2-i* discs induced for 5 days (**L**), and *ap* > *p35* + *GFP* + *POLA2-i+stg* discs induced for 5 and 10 days (**M** and **N**, respectively) stained with anti-PH3. Note that images in **K***–***M** were re-sized to match the scale bar in all panels. Magnifications of all discs are shown in **K***'–***N***'*. In the merge, DAPI, GFP, and PH3 are shown in blue, green, and grayscale, respectively. Scale bars, 100 µm. In this figure, *ap* > *p35* + *GFP* + *POLA2-i* discs with induction lengths of 4 or more days spent 7 days at 18 °C prior to induction.

We observed that larvae with discs experiencing RS but protected against apoptosis (POLA2-i + p35) had an extended larval period. This is a characteristic phenotype of *Drosophila* larvae with tumors [50]. This suggested that RS in cells resistant to apoptosis could suffice to induce oncogenic features. Consistent with this possibility, POLA2-i + p35 discs dissected and imaged 4 and 7 days after transgene induction appeared gradually disorganized (Fig. 7A–C). Tissue disorganization is a fundamental characteristic of tumors.

In most carcinomas, the progression toward malignancy involves a loss of epithelial integrity [51]. The integrity of epithelial architecture depends on the polarization of the plasma membrane into apical and basolateral domains. The Scribbled/Discs Large/Lethal Giant Larvae complex localizes to the basolateral region of the epithelium and plays central roles in maintaining epithelial polarity and normal development [51]. We utilized antibodies against Discs Large (Dlg) to assess potential defects in epithelial integrity. Control tissues showed Dlg enrichment in the basolateral region of the wing epithelium (Fig. 7D). In contrast, Dlg localized surrounding the cellular contours of POLA2-depleted cells and did not show a discrete localization as observed in normal cells (Fig. 7D–F). This indicates that POLA2 downregulation combined with suppression of apoptosis disrupts epithelial integrity, which can exacerbate cell motility and invasiveness, often considered a prerequisite for tumor infiltration and metastasis [51, 52]. In *Drosophila* development, cells from different compartments (e.g., dorsal and ventral) are prevented from mixing with cells from other compartments [53]. Interestingly, we observed in numerous discs GFP-positive cells that were mis-localized and were no longer clustered with the rest of the dorsal cells (Fig. 7E, F, H), suggesting that POLA2-i + p35 cells have acquired the potential to migrate and invade the surrounding tissue.

The JNK pathway regulates cell polarity and epithelial integrity, and JNK activation can promote tumorigenic behaviors in *Drosophila* [54]. We observed an abnormal upregulation of the JNK pathway in POLA2-i + p35 discs, as revealed by antibodies against the phosphorylated form of JNK (pJNK) and the JNK target gene MMP1 [55, 56] (Fig. 7H). Those antibodies were undetectable in control discs (Fig. 7G). These results confirm and extend previous research findings indicating that conditions compromising cell viability combined with suppression of apoptosis can promote tumor formation in the wing imaginal disc [35, 47, 57–59].

### Cdc25/string promotes tumor growth in wing discs with RS

A central hallmark of cancer is the ability to sustain proliferative capacity. Although POLA2-i + p35 discs exhibited oncogenic features, our cell cycle analysis revealed that cells in those discs accumulate in G2 (Fig. 4). Corroborating this, antibodies against the mitotic marker phospho-histone H3 (PH3) revealed that POLA2-i + p35 discs were seldom PH3-positive (Fig. 7K, L). A closer inspection of the rare mitotic cells found in those discs revealed the presence of mitotic defects such as lagging and misplaced chromosomes (Fig. 7I, J; Fig. S8). These are signs of chromosomal instability, which is a proposed cancer driver [60].

Limiting G2/M transition could serve as a mechanism to prevent tumorigenesis in cells with RS. We analyzed whether the induction of G2/M transition in the POLA2-i + p35 context could enhance tumor growth. The phosphatase cdc25/stg is a central driver of G2/M transition in eukaryotic cells [61]. Previously, we showed that tetraploid cells that arise after cytokinesis failure accumulated in G2, and that cdc25/stg upregulation bypassed the G2 arrest and drove tumorigenesis [62]. We found that cells overexpressing cdc25/stg in a context of POLA2 depletion formed tumorous discs (Fig. 7M, N). Notably, PH3-positive cells were frequently observed in those tumors (Fig. 7M, N), suggesting that cdc25/stg upregulation bypasses the G2 blockade and drives cell proliferation. These observations indicate that cdc25/stg, when upregulated in cells with RS unable to die by apoptosis, promotes tumor growth.

## Discussion

The results presented here reveal that POLA2 downregulation in vivo slows the rate of DNA replication and causes the accumulation of ssDNA, which are hallmarks of RS [4]. We find that cells with reduced POLA complex rely on dATR to limit the accumulation of DNA damage and sustain cell viability. Previous analyses in cell lines have also shown that ATR prevents the accumulation of DNA damage [63, 64]. However, the mechanisms leading to fork collapse when ATR activity is defective have yet to be determined. The prevailing hypothesis proposes that fork collapse and the formation of DSBs may be an active process mediated by the activation of one or more nucleases [63]. The in vivo model presented here provides a system that could be utilized to examine the roles of specific nucleases in the collapse of replication forks in cells with defective dATR signaling.

We have shown that cells with reduced POLA2 activate p53 and JNK. While these pathways contribute to the elimination of cells with RS, downregulation of p53 leads to a near total rescue of apoptosis. In contrast, JNK appears to play a more marginal role in this response. Previous studies demonstrated that p53 and JNK establish a feedback loop that amplifies the apoptotic response [65]. That mechanism might be used in cells with RS to reinforce the p53-dependent apoptotic response.

We reveal a crucial function of Lig4 in cells with RS. Studies in vitro have involved key proteins in NHEJ, including Lig4, in the response to RS. Those studies showed that cell lines defective in NHEJ exhibit enhanced sensitivity to DNA replication inhibitors [66–68]. Our observations, combined with those results, implicate NHEJ as a central player in protecting cells experiencing RS. While HR has previously been shown to play a key role in protecting mammalian cancer cells in culture from RS [69–72], much less is known about how NHEJ is activated under these conditions.

Apoptosis is key to suppress oncogenesis, and the ability to circumvent apoptosis is a hallmark of advanced cancers [73, 74]. We show here that cells with RS enter apoptotic programs as a protective mechanism to prevent tumorigenesis. Only when apoptosis is suppressed, cells with RS express oncogenic characteristics such as lack of epithelial integrity, activation of the oncogenic JNK pathway, chromosomal instability, and invasion. Although oncogenic activation induces RS and genomic instability during disease progression [75], our findings show that RS induced independently of oncogenic activation is sufficient to activate oncogenic programs when apoptosis is suppressed. Despite exhibiting oncogenic traits, these cells proliferate poorly and accumulate in G2. We find that upregulation of cdc25/stg can evade that barrier and enhances the oncogenic phenotype. In a previous study, we found that cytokinesis failure leads to G2 arrest and apoptosis, and suppression of apoptosis and upregulation of cdc25/stg promoted genomic instability and tumorigenesis [62]. Those observations, together with the results presented here, suggest a link between cytokinesis failure, RS, and tumorigenesis in vivo. In line with this, a recent study in human cell lines demonstrated that, after cytokinesis defects, cells undergo RS and that this is a major driver of genomic instability and tumorigenesis [76].

## Methods

### *Drosophila* strains

The following strains were obtained from the Bloomington *Drosophila* Stock Center (BDSC): *UAS-POLA2 RNAi* (77417), *UAS-Prim2 RNAi* (44584), *UAS-mCD8-GFP* (5137, GFP in the text), *UAS-p35* (5072, insertion in Chr2; and 6298, insertion in ChrX)*, UAS-bsk-DN* (6409), *UAS-dATR RNAi* (41934), *UAS-dATM RNAi* (44073), *UAS-stg* (56562), *act-Gal4* (4414), *gug-Gal4* (6773), and *tub-G80ts* (7018, insertion in Chr3; and 7019, insertion in Chr2). The following strains were obtained from the Vienna *Drosophila* Resource Center (VDRC): *UAS-Brca2 RNAi* (GD 39957), *UAS-Lig4 RNAi* (KK 107044), and *UAS-p53 RNAi* (KK 103001). Other strains used for this article were *ap-Gal4* (Calleja et al*.*, 1996)*, RPA1-GFP* (Blythe and Wieschaus, 2015), *TRE-RFP* (Chatterjee and Bohmann, 2012), *hid 5’F-GFP* (hid-GFP in the text; Tanaka-Matakatsu et al*.*, 2009), and the *yw* (*yellow white*) mutant strain. Some of these transgenes were combined into unique fly stocks: *ap-Gal4,UAS-mCD8-GFP/CyO; tub-G80ts/TM6B, ap-Gal4,UAS-mCD8-GFP,UAS-p35/CyO; tub-G80ts, ap-Gal4,tub-G80ts/CyO; UAS-POLA2 RNAi, ap-Gal4,UAS-mCD8-GFP/CyO; UAS-POLA2 RNAi,tub-G80ts*, *act-Gal4/SM5a-TM6B/tub-G80ts*, and *gug-Gal4,tub-G80ts/TM6B*.

### Cross maintenance

Crosses were stored at 18 °C in standard conditions, flipped every 2 or 3 days to allow egg laying, and moved to 29 °C to induce Gal4 expression. For an induction length of 4 days, vials were induced right after flipping. For an induction length of 2 days, vials were maintained at 18 °C for an extra 5–7 days after flipping before induction. For irradiated samples, refer to the section below. Any other modification to this protocol is specified in the corresponding figure legend.

### Treatment with ionizing radiation

Vials were maintained at 18 °C for around 8–10 days from the date of flipping. They were then switched to 29 °C to drive transgene expression 24 h prior to irradiation. Irradiation was carried out in a Xylon MaxiShot X-ray machine at a dose of 40 Gy. After irradiation, samples were maintained at 29 °C for 4 h before dissection.

### Immunofluorescence

Primary antibodies were acquired from Developmental Studies Hybridoma Bank (DSHB), Cell Signaling Technology (CST), Rockland Sciences, Promega, and Abcam. Primary antibodies at the indicated dilutions were employed, as follows: mouse anti-pH2Av (DSHB, no. UNC93-5.2.1, 1:500), mouse anti-Dlg (DSHB, no. 4F3 anti-discs large, 1:100), mouse anti-MMP1 (DSHB, no. 5H7B11/3A6B4/3B8D12 in a 1:1:1 mixture, 1:50), rabbit anti-Dcp1 (CST, no. 9578, 1:100), rabbit anti-PH3 (CST, no. 9701, 1:100), rabbit anti-pH2Av (Rockland, no. 600-401-914, 1:1000), rabbit anti-pJNK (Promega, no. V7931, 1:100), and chicken anti-GFP (Abcam, no. ab13970, 1:1000). The rabbit anti-Rad51 primary antibody (1:1000) was a kind gift from Dr Irene Chiolo (University of Southern California). Secondary antibodies were acquired from Invitrogen. All of them were generated in goat and used at a 1:400 dilution: anti-mouse IgG-488 (no. A-11017), anti-mouse IgG-555 (no. A-21425), anti-rabbit IgG-555 (no. A-21430), anti-rabbit IgG-647 (no. A-21246), and anti-chicken IgY-488 (no. A-11039). 4’,6-diamino-2-phenylindole (DAPI) was obtained from Invitrogen (no. D1306) and used at a 600 nM concentration.

Wing imaginal discs from third instar wandering larvae were dissected in cold phosphate-buffered saline (PBS) and fixed in 3.7% formaldehyde diluted in PBS for 20 min at room temperature (RT). Samples were washed three times for 10 min in 0.2% Triton X-100 diluted in PBS (PBT) and then blocked for at least 20 min in 0.5–1% BSA diluted in PBT with 250 mM NaCl (BBT). Following this, they were incubated in primary antibody diluted in BBT at RT overnight or at 4 °C for around 60 h. Samples were washed three times for 10 min in BBT and were incubated with DAPI and fluorescent secondary antibodies for 1.5 h at RT before being washed four times for 15 minutes in PBT. Wing imaginal discs were mounted in 90% glycerol in PBS containing 0.5% propyl gallate and maintained at 4 °C.

### EdU staining

This protocol was adapted from the Click-iT EdU Cell Proliferation Kit for Imaging (no. C10340) from Invitrogen. Wing imaginal discs were dissected in cold PBS and incubated with 300 µM 5-ethynyl-2’-deoxyuridine (EdU) (Sigma-Aldrich, no. 900584) for 30 min at RT. Samples were then fixed in 3.7% formaldehyde diluted in PBS for 20 min at RT, washed three times for 10 min in PBT and blocked for at least 20 min in BBT. If applicable, samples were incubated with primary antibodies diluted in BBT at RT overnight or at 4 °C for around 60 h. After that, samples were then washed three times for 10 min in BBT and incubated in the Click-IT reaction solution for 1 h at RT. Samples were then washed once for 10 min in BBT, stained with DAPI and fluorescent secondary antibodies for 1.5 h at RT, and washed four times for 15 min with PBT. Wing imaginal discs were mounted in 90% glycerol in PBS containing 0.5% N-propyl gallate and maintained at 4 °C.

### RNA extraction, cDNA synthesis and quantitative PCR

Total RNA from 2 to 4 whole third instar wandering larvae was extracted as per the manufacturer’s instructions with the RNeasy Mini Kit (Qiagen, no. 74106) and then treated with RQ1 RNase-free DNase (Promega, no. M6101). RNA samples were converted to cDNA with the SuperScript III Reverse Transcriptase (Invitrogen, no. 18080-044). Quantitative PCR was performed on the QuantStudio 6 Flex Real-Time PCR System (Applied Biosystems). Each sample was run in triplicates. Reaction mixes contained 5 ng of cDNA, 400 nM of forward and reverse primers each, and 1x HOT FIREPol EvaGreen qPCR Mix Plus (Solis Biodyne, no. 08-24-00001). Primers were obtained from TAG Copenhagen and their sequences are shown below:

RP49 forward: AAGCGGCGACGCACTCTGTT

RP49 reverse: GCCCAGCATACAGGCCCAAG

POLA2 forward: ACGATCCCATGCTGGATGAC

POLA2 reverse: TGCCGATTGGGGTGTATAGC

### Flow cytometry

Wing imaginal discs from 15 to 20 third instar wandering larvae were dissected in cold PBS and immediately incubated in a solution of 0.1% trypsin-EDTA diluted in PBS for 30 min at 32 °C to enzymatically digest the tissue. Then, the mixture was pipetted up and down with force with a P1000 to mechanically digest the tissue. Trypsin was inactivated with fetal bovine serum and then samples were fixed in 3.7% formaldehyde at RT for 20 min followed by a cell permeabilization with 70% ethanol at RT for 1 h. Afterwards, samples were incubated at RT for 1 h in a solution of DAPI 1 µg/mL and RNase A 200 µg/mL diluted in PBS with 0.1% Triton X-100. Lastly, samples were re-suspended in PBS and filtered through a cell strainer. DAPI and GFP fluorescence were measured in a BD FACSAria Fusion flow cytometer, and data was analyzed with the FlowJo software. GFP-positive single cells were selected and histogram plots for DAPI signal and forward scatter values were obtained to assess DNA content and cellular size, respectively.

### Image processing and data analysis

Images were obtained using a Leica SP8 confocal microscope and processed with the Fiji software. The exact procedures for quantification are explained in the sections below. Figures were prepared with Adobe Illustrator.

### Quantification of EdU intensity and EdU-positive cells

Confocal images of the wing pouch of wing imaginal discs labeled with EdU were taken with the Leica SP8 confocal microscope and uploaded to Fiji. The dorsal area (GFP-positive) was selected and uploaded to the ROI manager. To quantify EdU intensity, a threshold was applied on the EdU channel to select the EdU-positive signal. The mean intensity of the EdU signal within the selected area was determined by using the “measure” function within the ROI manager. To quantify EdU-positive cells, the plugin “Cell Counter” was used to count all EdU-positive cells within the selected dorsal area, which was also measured. Results were expressed as the number of EdU-positive cells per 100 µm^2^.

### Quantification of Rad51 foci

Confocal images of the wing pouch of wing imaginal discs were taken with the Leica SP8 confocal microscope and uploaded to Fiji. The “polygon” selection tool was used to select an area of cells (DAPI-positive) from the dorsal compartment (GFP- or pH2Av-positive). The selected area was measured and the Rad51 foci were counted with the “Find Maxima” tool. Results were expressed as number of Rad51 foci per 1000 µm^2^.

### Quantification of dorsal compartment size

Confocal Z-stack images of wing imaginal discs were taken with the Leica SP8 confocal microscope. These images were uploaded to Fiji and a maximum intensity projection was applied. For samples labeled with GFP to mark the dorsal compartment, a “median” of 5 was used on the GFP channel and a threshold was set to select the GFP-positive tissue. The selection was added to the ROI manager and the dorsal area was determined with the “measure” option within the ROI manager. For samples labeled with pH2Av to mark the dorsal compartment, the pH2Av-positive tissue was selected with the “polygon” selection tool. The selection was added to the ROI manager and the dorsal area was determined with the “measure” option within the ROI manager. Results were expressed as the fold change of dorsal area relative to the control sample.

### Quantification of apoptotic area

Confocal Z-stack images of wing imaginal discs were taken with the Leica SP8 confocal microscope. These images were uploaded to Fiji and processed as explained in the previous section to select the dorsal compartment through the GFP or pH2Av labels. A “median” of 1 was applied to the Dcp1 channel and a threshold was applied to select the Dcp1-positive signal. The area of the Dcp1 signal within the dorsal compartment was determined by using the “measure” function within the ROI manager. The Dcp1 area was normalized to the already measured dorsal compartment area. Results were expressed as the fold change of Dcp1 area/dorsal area relative to the control sample.

### Statistics

Graphs and statistical analyses were performed with GraphPad Prism. Data is always presented as mean ± SD, and individual data points are also plotted into the graphs. The number of biological replicates quantified per sample, the exact statistical tests performed, and the obtained p-values are indicated in the corresponding figures and figure legends. All experiments were replicated at least once. No data was excluded from the analyses. Researchers were not blinded during the data analysis process.

## Supplementary information

Supplementary Figures

## Data Availability

All data generated or analyzed during this study are included in this published article and its supplementary information file.
