# Supplementary material for: Replication stress promotes cellular transformation in Drosophila epithelium
Source: Cell Death Discov. 2025 Mar 12;11:96. doi: 10.1038/s41420-025-02383-2 (PMC11904189; doi:10.1038/s41420-025-02383-2)
Supplement: Supplementary file 1 — Supplementary Figures [file 41420_2025_2383_MOESM1_ESM.docx]

**SUPPLEMENTARY FIGURES**

**
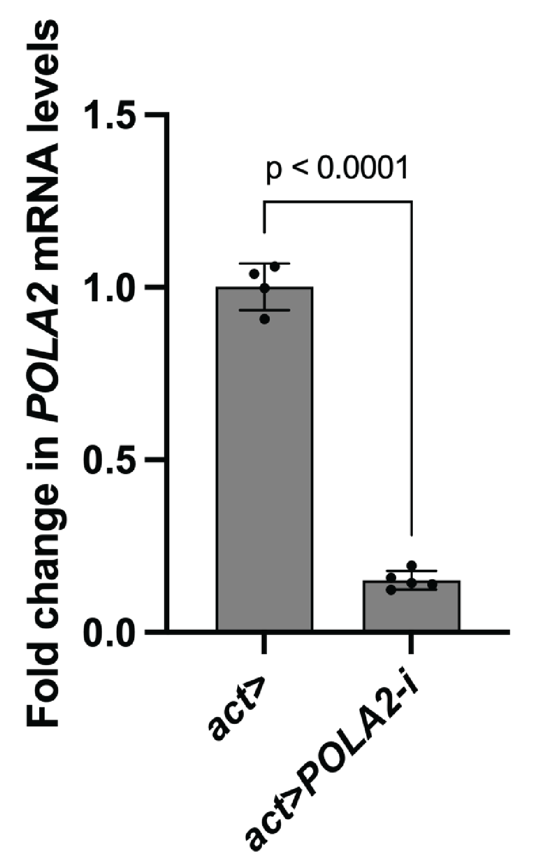
**

**Figure S1. Efficiency of *UAS-POLA2 RNAi***

Quantification of *POLA2* mRNA levels in whole larvae expressing *POLA2-i* for 4 days under *act-Gal4* control (*act>POLA2-i*; n = 5) represented as a fold change in mRNA levels relative to control larvae (*act>;* n = 4). Data shown are mean ± SD. Statistical analysis was performed using a two-tailed unpaired t-test.

**
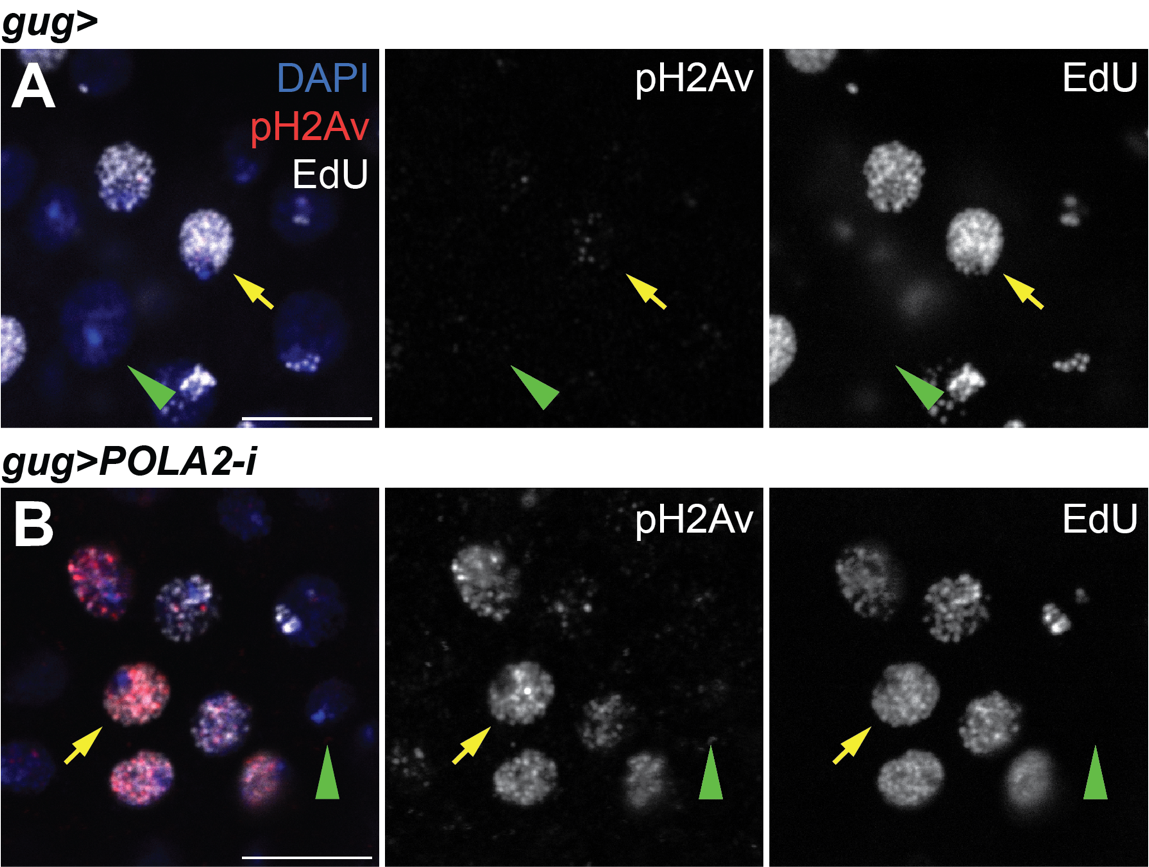
**

**Figure S2. Replication Checkpoint Activation in the Peripodial Membrane**

(A, B) Confocal images of the peripodial membrane of control (*gug>*) (A) and POLA2-downregulating (*gug>POLA2-i*) (B) discs stained with anti-pH2Av and EdU. The *gug-Gal4* driver was used to drive transgene expression in the peripodial membrane for 2 days. Yellow arrows point to EdU-positive cells and green arrowheads point to EdU-negative cells. In the merge, DAPI, pH2Av, and EdU are shown in blue, red, and grayscale, respectively. Scale bars, 10 µm.

**
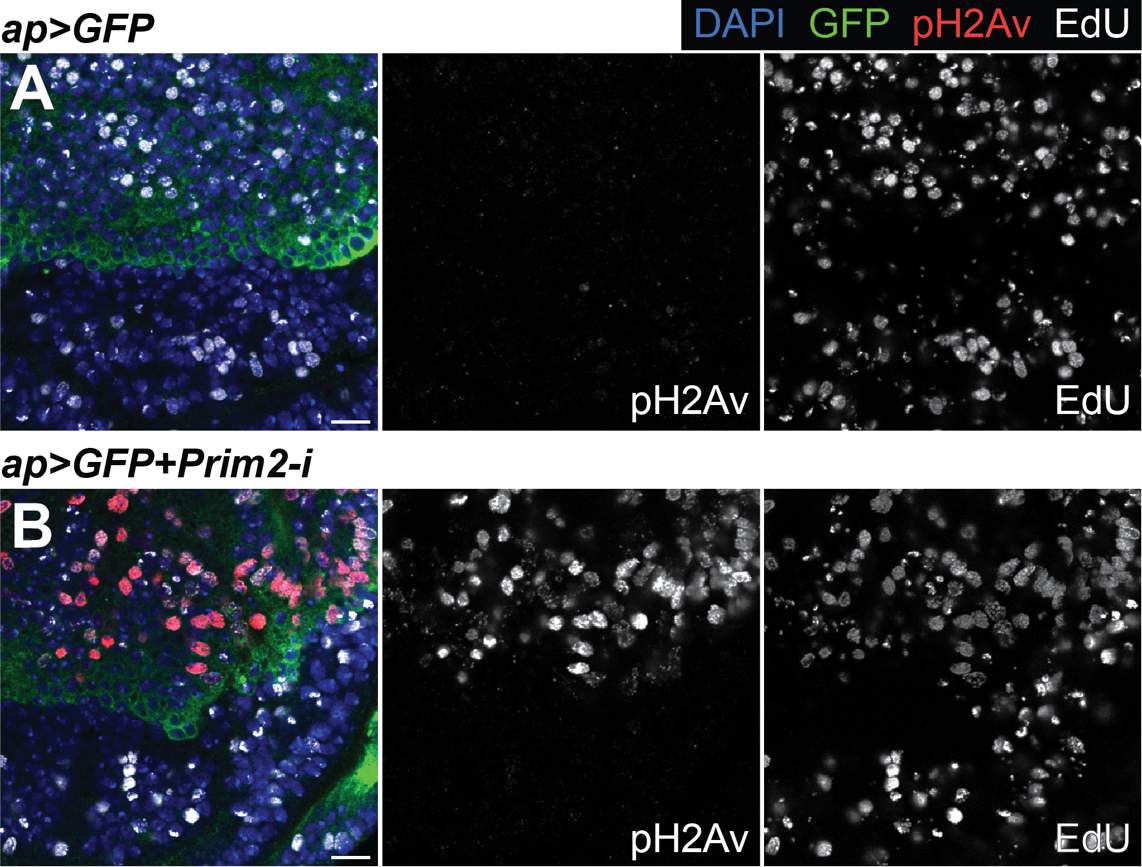
**

**Figure S3. Prim2 Depletion Causes RS in the Wing Imaginal Disc**

(A, B) Confocal images of the wing pouch of *ap>GFP* (A) and *ap>GFP+Prim2-i* (B) discs induced for 2 days and labeled with anti-pH2Av and EdU. In the merge, DAPI, GFP, pH2Av, and EdU are shown in blue, green, red, and grayscale, respectively. Scale bars, 10 µm.

**
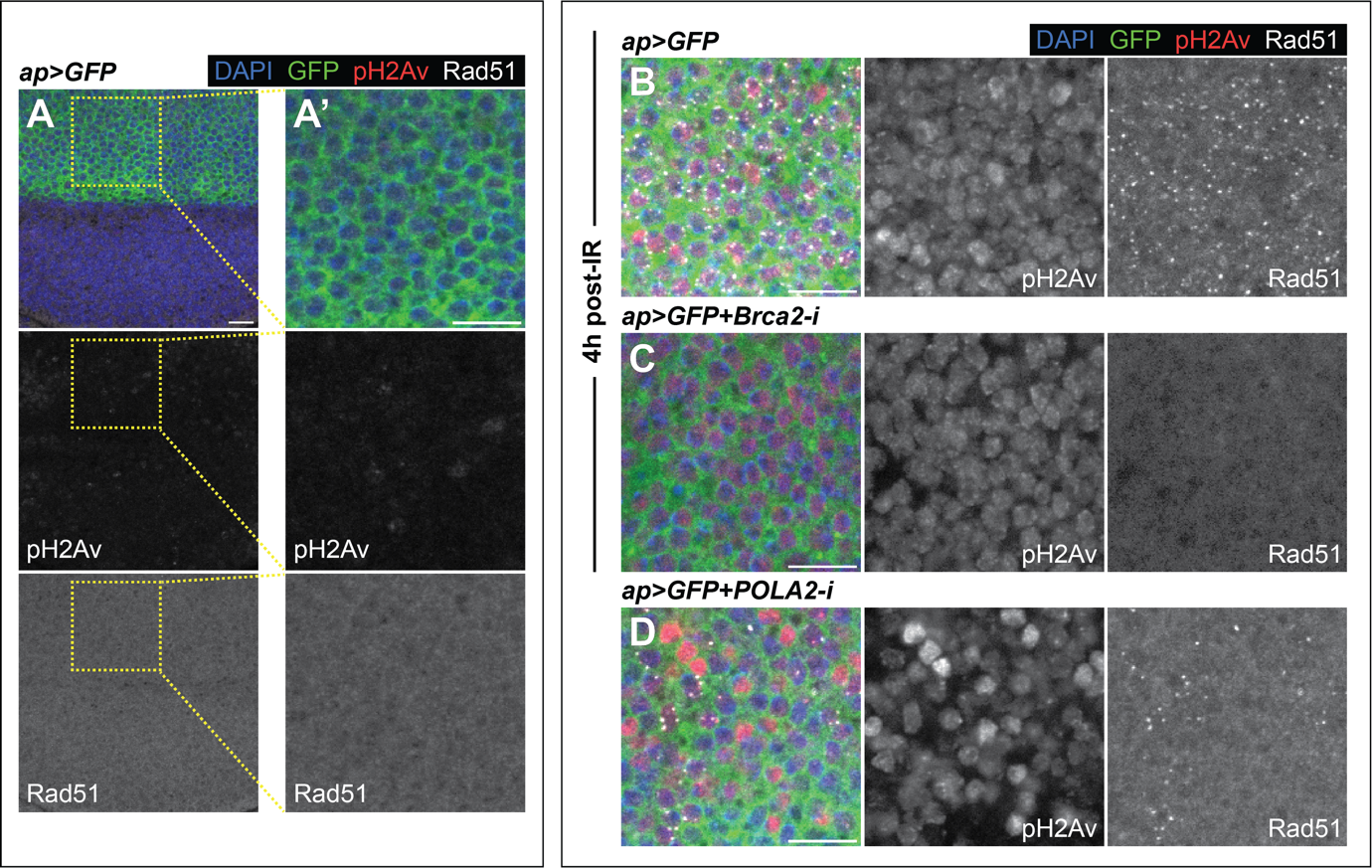
**

**Figure S4. POLA2 Downregulation Causes Checkpoint Activation but Reduced DNA Damage in Wing Imaginal Discs**

(A) Confocal image of the wing pouch of an *ap>GFP* disc stained with anti-pH2Av and anti-Rad51. A magnification of the dorsal compartment is shown in *A’.* In the merge, DAPI, GFP, pH2Av, and Rad51 are shown in blue, green, red, and grayscale, respectively. Scale bars, 10 µm.

(B-D) Confocal images of the dorsal compartment within the wing pouch of *ap>GFP* (B) and *ap>GFP+Brca2-i* (C) discs 4 hours after irradiation and of a non-irradiated *ap>GFP+POLA2-i* (D) disc stained with anti-pH2Av and anti-Rad51. These images are complementary to those in Fig 2D-F but represent different discs. In the merge, DAPI, GFP, pH2Av, and Rad51 are shown in blue, green, red, and grayscale, respectively. Scale bars, 10 µm.

**
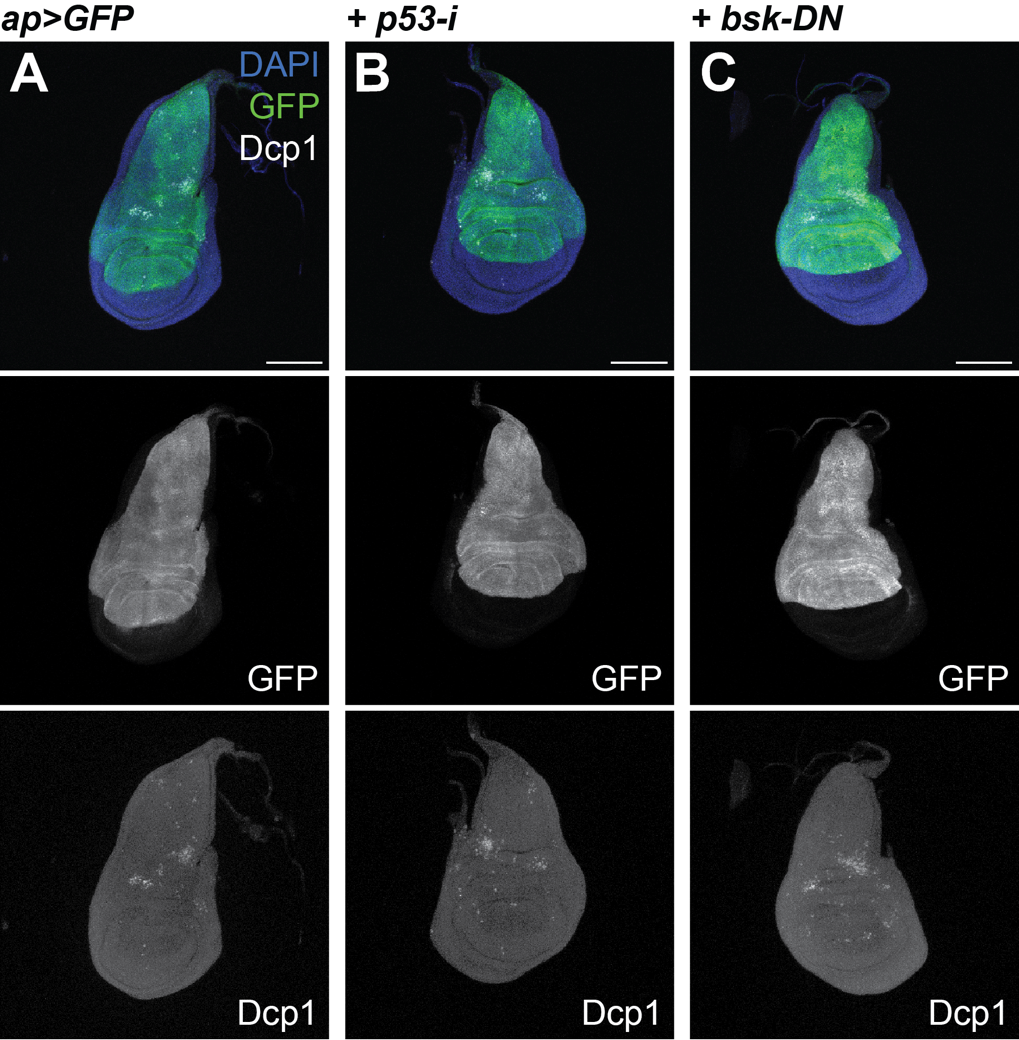
**

**Figure S5. p53 and JNK Downregulation in Wing Imaginal Discs**

(A-C) Confocal images of *ap>GFP* (A), *ap>GFP+p53-i* (B), and *ap>GFP+bsk-DN* (C) discs stained with anti-Dcp1. In the merge, DAPI, GFP, and Dcp1 are shown in blue, green, and grayscale, respectively. Scale bars, 100 µm.

**
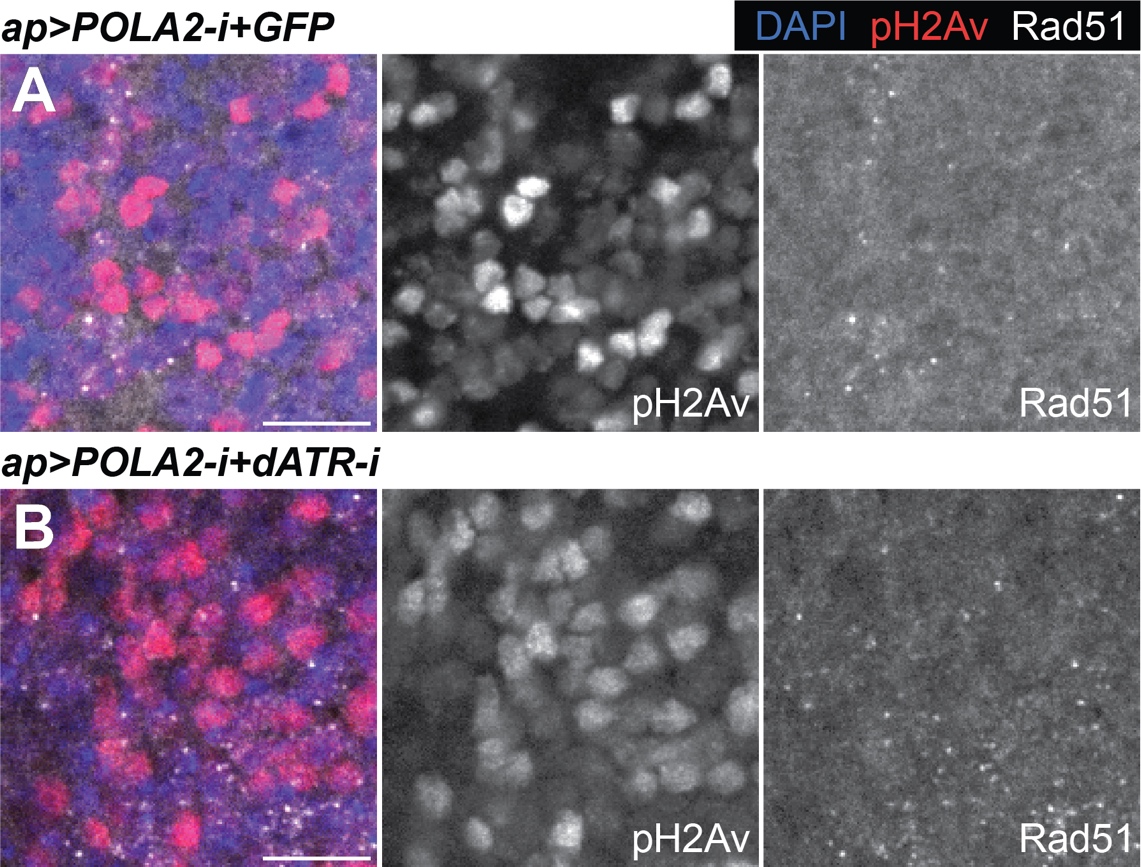
**

**Figure S6. dATR Prevents the Formation of DSBs in POLA2-Depleted Tissues**

(A, B) Confocal images of the dorsal compartment within the wing pouch of *ap>POLA2-i+GFP* (GFP not shown) (A) and *ap>POLA2-i+dATR-i* (B) discs labeled with anti-pH2Av and anti-Rad51. These images are complementary to those in Fig 5I, J but represent different discs. In the merge, DAPI, pH2Av, and Rad51 are shown in blue, red, and grayscale, respectively. Scale bars, 10 µm.

**
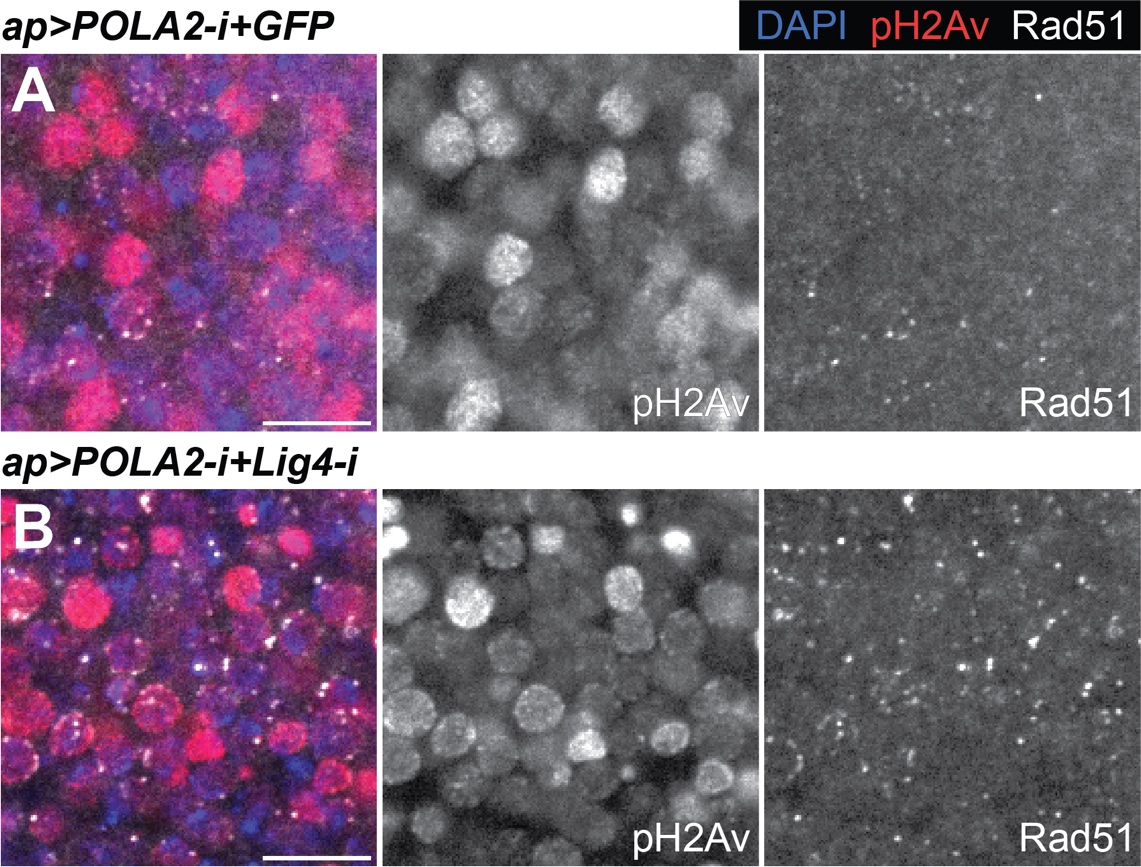
**

**Figure S7. Lig4 Prevents the Formation of DSBs in POLA2-Depleted Tissues**

(A, B) Confocal images of the dorsal compartment within the wing pouch of *ap>POLA2-i+GFP* (GFP not shown) (A) and *ap>POLA2-i+Lig4-i* (B) discs labeled with anti-pH2Av and anti-Rad51. These images are complementary to those in Fig 6I, J but represent different discs. In the merge, DAPI, pH2Av, and Rad51 are shown in blue, red, and grayscale, respectively. Scale bars, 10 µm.

**
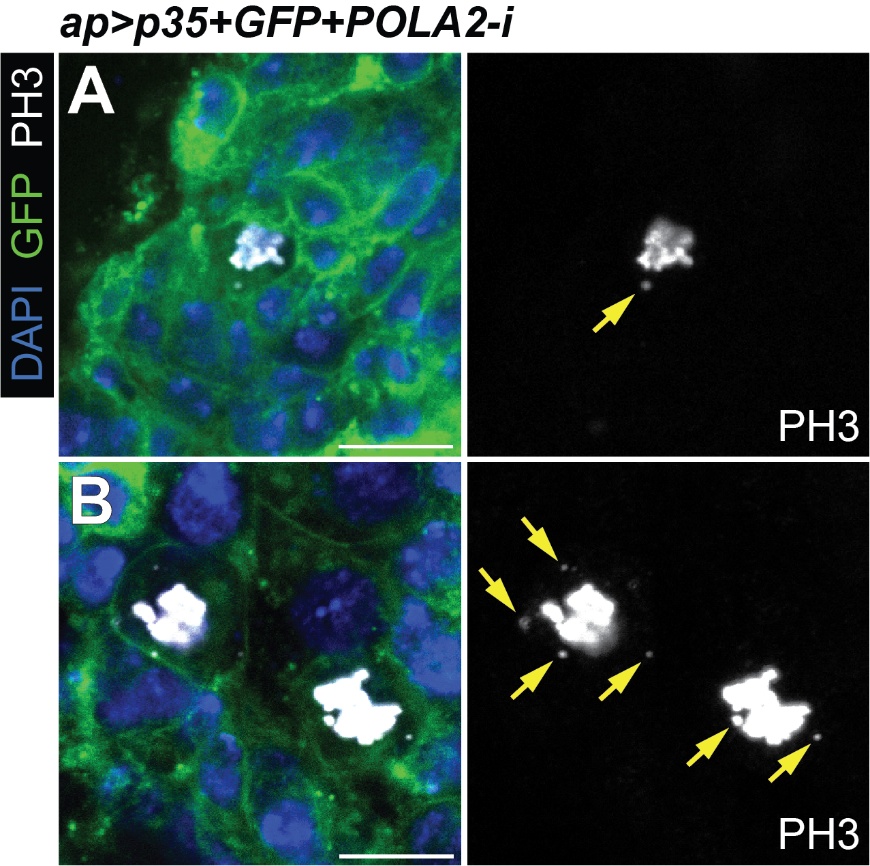
**

**Figure S8. Aberrant Mitoses in POLA2-depleted Discs Where Apoptosis is Inhibited**

(A, B) Confocal images of mitotic cells with misplaced chromosomes (yellow arrows) stained with anti-PH3 in *ap>p35+GFP+POLA2-i* discs induced for 5 days. Note that these discs spent 7 days at 18°C prior to induction. In the merge, DAPI, GFP, and PH3 and shown in blue, green, and grayscale, respectively. Scale bars, 10 µm.
